# Untangling the KRAS mutated lung cancer subsets and its therapeutic implications

**DOI:** 10.1186/s43556-021-00061-0

**Published:** 2021-12-17

**Authors:** Kulshrestha Ritu, Pawan Kumar, Amit Singh, K. Nupur, Sonam Spalgias, Parul Mrigpuri, Rajkumar

**Affiliations:** 1 Department of Pathology, V.P.Chest Institute, University of Delhi, New Delhi, 110007 India; 2 Department of Pulmonary Medicine, V.P.Chest Institute, University of Delhi, New Delhi, India

**Keywords:** Lung cancer, KRAS mutation, KRAS- signaling pathway, KRAS targeted therapies

## Abstract

The Kirsten rat sarcoma virus transforming protein (KRAS) mutations (predominate in codons 12, 13, and 61) and genomically drive nearly one-third of lung carcinomas. These mutations have complex functions in tumorigenesis, and influence the tumor response to chemotherapy and tyrosine kinase inhibitors resulting in a poorer patient prognosis. Recent attempts using targeted therapies against KRAS alone have met with little success. The existence of specific subsets of lung cancer based on KRAS mutations and coexisting mutations are suggested. Their interactions need further elaboration before newer promising targeted therapies for KRAS mutant lung cancers can be used as earlier lines of therapy. We summarize the existing knowledge of KRAS mutations and their coexisting mutations that is relevant to lung cancer treatment, in this review. We elaborate on the prognostic impact of clinical and pathologic characteristics of lung cancer patients associated with KRAS mutations. We briefly review the currently available techniques for KRAS mutation detection on biopsy and cytology samples. Finally, we discuss the new therapeutic strategies for targeting KRAS-mutant non-small cell lung cancer (NSCLC). These may herald a new era in the treatment of KRAS^G12C^mutated NSCLC as well as be helpful to develop demographic subsets to predict targeted therapies and prognosis of lung cancer patients.

## Introduction

Lung cancer is the leading cause of cancer-related deaths among males worldwide [1]. It accounts for 1.38 million cancer deaths per year. It is the fifth common cause of cancer among females [2]. The overall 5-year survival rate of lung cancer remains poor in spite of numerous recent advances in its detection and treatment [3]. Identifying the molecular subsets of lung adenocarcinoma (LADC) and personalized treatment with targeted therapy, is needed to improve patient prognosis and survival [4]. Recent studies have highlighted the need to identify sub-sets of co-existing mutations in the EGFR-mutated LADC, as these may have a major impact on prognosis and newer therapeutic approaches [5].

In lung adenocarcinomas, comprehensive molecular profiling has identified significant mutations in eighteen genes [5], including (Table 1): (i) Oncogenes; EGFR (20–50%) [5, 6, 36], KRAS (33%) [7], BRAF (10%) [8], PI3K (7%), MET (7%) [13], RIT1 (2%), NRB1 [36],ERBB2 [16](ii) tumour suppressor genes; TP53 (46%) [19], STK11(17%), KEAP1(17%), NF1(11%), SETD2(9%), ARID1A(7%),RB1 (4%), CDKN2A (4%), (iii) Gene fusions/splice site mutations causing aberrant RNA transcripts: EML4-ALK [32], CD74-ROS1 [31], KIF5B-RET [33], NTRK [34] and NRG1fusions [35].

**Table 1:** Significant mutations identified by comprehensive molecular profiling in lung adenocarcinoma

Oncogenes (Chromosome Location)	Mutations seen	Reference
**Oncogenes**		
EGFR (7)	Common in exons 18–21, Amplifications, deletions, point mutations at T790M, G719X, L858 etc., Rare in exons 6, 7, 8, 12, 15, and 17	[5, 6]
KRAS (12p12.1)	exon 2 and exon 3 codons 12, 13, and 61	[7]
BRAF (7q34)	exon 15; glutamate substitution for valine at codon 600 (V600E) and non-V600Emutations(activating-G469A/V, K601E, L597R) or (inactivating- D594G, G466V)	[8–10]
*PIK3CA* (3q26.32)	20 hotspot regions in exon 9 and exon 20	[11, 12]
*MET* (7q31.2)	exon 14 skipping mutations, Splice	[13]
*RIT1* (1q22)	Exons 1–6 substitutions	[14]
*NRB1* (7q21.3)	Neurabin 1	[15]
*ERBB2/ HER2* (17q12)	Amplifications, intragenic insertions	[16]
HRAS (11p15.5)	codons 12 and 13	[17]
NRAS (1p13.1)	Mutations which change amino acid residues 12, 13 or 61	[18]
**Tumour Suppressor Genes**		
TP53 (17p13.1)	C > A transversions in the TP53 gene	[19–21]
STK11 (19p13.3)	high expression in the testis and fetal liver	[22, 23]
KEAP-1 (19p13.2)	key sensor of oxidative and electrophilic stress	[24, 25]
NF1 (17)	Truncation	[26]
RB-1 (13q14.2)	responsible for a major G1 checkpoint	[27]
CDKN2A (9p21.3)	Exons-1β, 1α, 2, and 3 that synthesize the proteins- p16 and p14ARF.	[28]
ARID1A (1p36.11)	key member of SWI/SNF chromatin-remodeling complex	[29]
SETD2 (3p21.31)	Loss of striatal neurons (Huntington’s disease)	[30]
PTEN (10q23.31)	Cowden Syndrome	[31]
**Fusion Oncogenes**		
EML4-ALK (2p23.2)	Responsible for 3–5% of NSCLC	[32]
CD74-ROS1 (6q22.1)	Rearrangement, Fusion	[31]
KIF5B-RET (10q11. 2)	Fusion	[33]
NTRK1/2/3-*NRG1* (1q23.1)	Fusion	[34, 35]

Patients with newly diagnosed lung adenocarcinoma commonly undergo sequential molecular testing (for EGFR, ALK, ROS1). They then undergo treatment with EGFR tyrosine kinase inhibitors- erlotinib, gefitinib, etc) and ALK/ROS1 TKIs (crizotinib, ceritinib) [37].KRAS mutations variably occur in LADC in western countries (20–25%) [38, 39] and in Asia (10–15%) [40, 41]. The identification of lung cancer patient subsets based on KRAS mutation analysis before initiation of EGFR targeted therapy needs to be done [42].

KRAS mutations predominantly occur in codon 12, 13 in lung cancer. Codons 10, 61 and 146 are much less frequently mutated. The prevalence of KRAS mutations in early and advanced stage LADCs is similar [7]. A heterogeneous spectrum of KRAS mutations; transversions (80%) or transitions (20%) [43] are identified in lung cancer patients. Patients with transversions, more frequently develop adenocarcinoma while those with transitions more frequently have squamous cell carcinoma [44]. Most KRAS mutations patients are males (60%), current or former smokers (63% and 33%, respectively) with adenocarcinoma (80%) [43]. KRAS mutations are rarely present in small cell lung cancer [45, 46]. The KRAS mutated LADCs grow in a solid pattern with TTF1 positivity (thyroid transcription factor) while the mucinous adenocarcinoma histology lacks TTF-1 [47]. The KRAS mutations are predictive of (i) poor prognosis [48] (ii) resistance to EGFR-TKI therapy in advanced cases [49] (iii) Exclusion of the EGFR and the BRAF mutations [50]. Thus, emphasizing the need to evaluate for KRAS and other coexisting mutations before the initiation of anti-EGFR therapy [51] (Table 1).

**Fig. 1:**
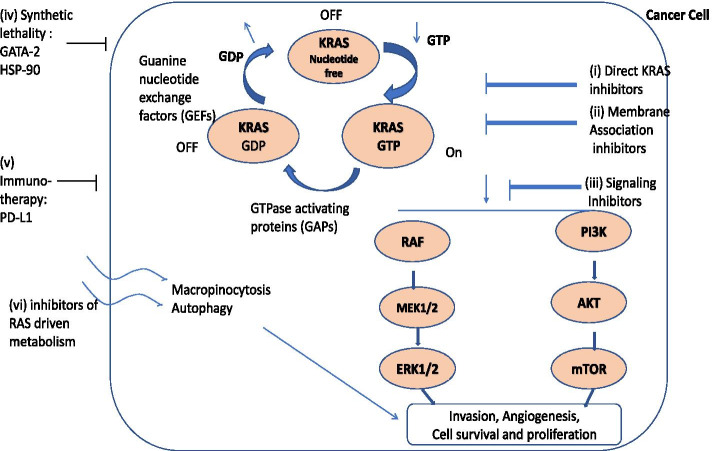
Mechanism of targeted action of therapeutic agents against KRAS driven carcinomas (i) Direct KRAS inhibitors- targets the RAS proteins activation and prevents the conversion of inactive KRAS to active KRAS (ii) KRAS membrane association - impairs KRAS post-translational modification, lipidation and localization (iii) KRAS downstream signaling pathways- inhibit downstream effector pathways- RAF, MEK, PI3K, mTOR (iv) KRAS synthetic lethality- selective killing of KRAS-mutant cells through inhibition of a second protein (v) Immunotherapy**-** immune checkpoint inhibitor therapy-inhibit PD-L1 (vi) Inhibition of RAS-regulated metabolic processes- targets mutant KRAS-driven metabolic rewiring

Molecular heterogeneity is observed in up to one-third of KRAS-mutant lung cancers and defines their chemotherapy response, tissue spread and prognosis [52]. The co-occurrence of two active mutations either drives oncogenes to functional redundancy [53] or results in cell senescence or death. Smokers with lung adenocarcinomas have concurrent KRAS [54], TP53, STK11, KEAP1 mutations while non-smokers with LADCs commonly have EGFR, TP53 mutations and/or MET alterations [5]. These subsets are associated with varied immune cell restrictions, altered angiogenesis, tumor microenvironment and poor survival [7, 39, 55]: (i) TP53 co-mutations activate the NF-κB pathway [56], increase IFNγ and PD-L1 expression [22] and promote an inflamed tumor immune microenvironment. (ii) *LKB1/*STK11co-mutations result in infiltration of neutrophils, leading to a pro-inflammatory cytokine milieu [22, 23]. (iii) KEAP1 mutations reduce T and B-lymphocytes infiltration [24] and NRF2 stabilization [24, 25]. (iv) Oncogenic MYC helps in immune evasion by KRAS-driven lung adenocarcinomas. By facilitating (a) IL-23-mediated expulsion of innate immune cells (T, B lymphocytes and NK cells), (b) CCL9-mediated macrophage recruitment and (c) VEGF mediated immunosuppressive microenvironment [57]. (v) PI3KCA co-mutations benefit activation of the BRAF pathway without risk of inducing senescence [58–60]. These studies have suggested that targeting the co-mutations and their pathways could be an effective treatment strategy in NSCLC patients [11, 12] (Table 2, Fig. 1).

**Table 2:** Molecular tests for KRAS detection

Method/Sensitivity (%)	Genes Detected	References
Sanger Sequencing (Gold Standard) (10–30%)	It detects variations in Codons, including base substitutions, insertions and deletions.	[61]
Whole Exome Sequencing	It can identify 18 statistically significant mutated genes	[36, 62]
Pyrosequencing (≤5%)	It is a sensitive method to detect the mutant *KRAS* alleles from paraffin-embedded tissue	[63]
PCR amplification with HRM analysis (10–20%)	It is used as a prescreening diagnostic method to detect mutations in KRAS, BRAF, PIK3CA, and AKT1	[64]
Allele-specific PCR (1–5%)	It uses ARMS and Scorpion probe technology to detect point mutations	[65]
SNaP Shot assay	It is a sensitive assay to detect mutant alleles in tumour cells (1%- 10% of total nucleated cells).	[66]

Previous studies using RNASeq data from The Cancer Genome Atlas have identified three subsets of KRAS mutated lung adenocarcinomas based on their dominant co-existing mutations. The three major subsets identified include; KL, KP and KC. These show co-occurring mutations in LKB1/ STK11 (KL), TP53 alterations (KP), CDKN2A/CDKN2B (KC). These biologically distinct subsets have unique intracellular signaling patterns and are susceptible to different therapeutic strategies [7]. KRAS alleles showed enrichment for KRASG12D in the KC subgroup. KL subsets showed enhanced sensitivity to several Hsp90 inhibitor drugs such as ganetespib appeared particularly effective [7] (Table 3).

**Table 3:** KRAS mutation directed lung cancer therapies

Mechanism of Action	Examples	Reference
KRAS membrane associations	Farnesyltransferase inhibitors (FTIs; tipifarnib, lonafarnib, salirasib) PDEδ inhibitors (Deltarasin)	[67–71]
Downstream effector signaling pathways	Single agent therapies; BRAF inhibitor (Sorafenib), MEK inhibitors (Selumetinib), mTOR inhibitor (ridafarolimus), focal adhesion kinase inhibitor (defactinib) Hsp90 inhibitor, ganetespib Combination therapies; PI3K inhibitor with MEK1/2 inhibitor (MEK162)	[7, 11, 72–76]
KRAS synthetic lethality	GATA2 inhibitor, (bortezomib) CDK-4 ablation TBK1, STK33 and PLK1 inhibition	[77–81]
Direct targeting of KRAS	Direct KRAS^G12C^ inhibitors, (Sotorasib and adagrasib)	[82]
Immunotherapywith Check point inhibitors	PD-L1 inhibitor- Pembrolizumab	[83]

## RAS family and downstream signaling

The RAS family of protooncogenes includes three isoforms; Kirsten rat sarcoma virus oncogene (KRAS) (chromosome 12p12.1), Harvey rat sarcoma virus (HRAS) (11p15.5), Neuroblastoma Ras sarcoma virus (NRAS) (1p13.1) [84]. KRAS (85%) is the predominant isoform followed by NRAS (11%) and HRAS (4%). These RAS genes encode a small membrane-localized guanosine triphosphate (GTP)-binding protein with intrinsic GTPase activity. Wild-type RAS proteins exist in an inactive state (GDP-bound) on the plasma membrane. They regulate the protein conformational change between active (GTP bound) and inactive states [85]. This process is regulated by; (i) Guanine Exchange Factors (that promote GDP dissociation and GTP binding), (ii) GAPs-GTPase activating proteins (that stimulate RAS GTPase activity). On mitogenic stimulation, the GEFs recruited to RAS, release GDP and form a transient nucleotide free state (Fig. 1). This nucleotide exchange causes conformational changes in RAS proteins (Switch 1 and Switch 2), which then bind to GTP, engage RAS effector proteins and activate RAS targets (Fig. 1).

**Fig. 2:**
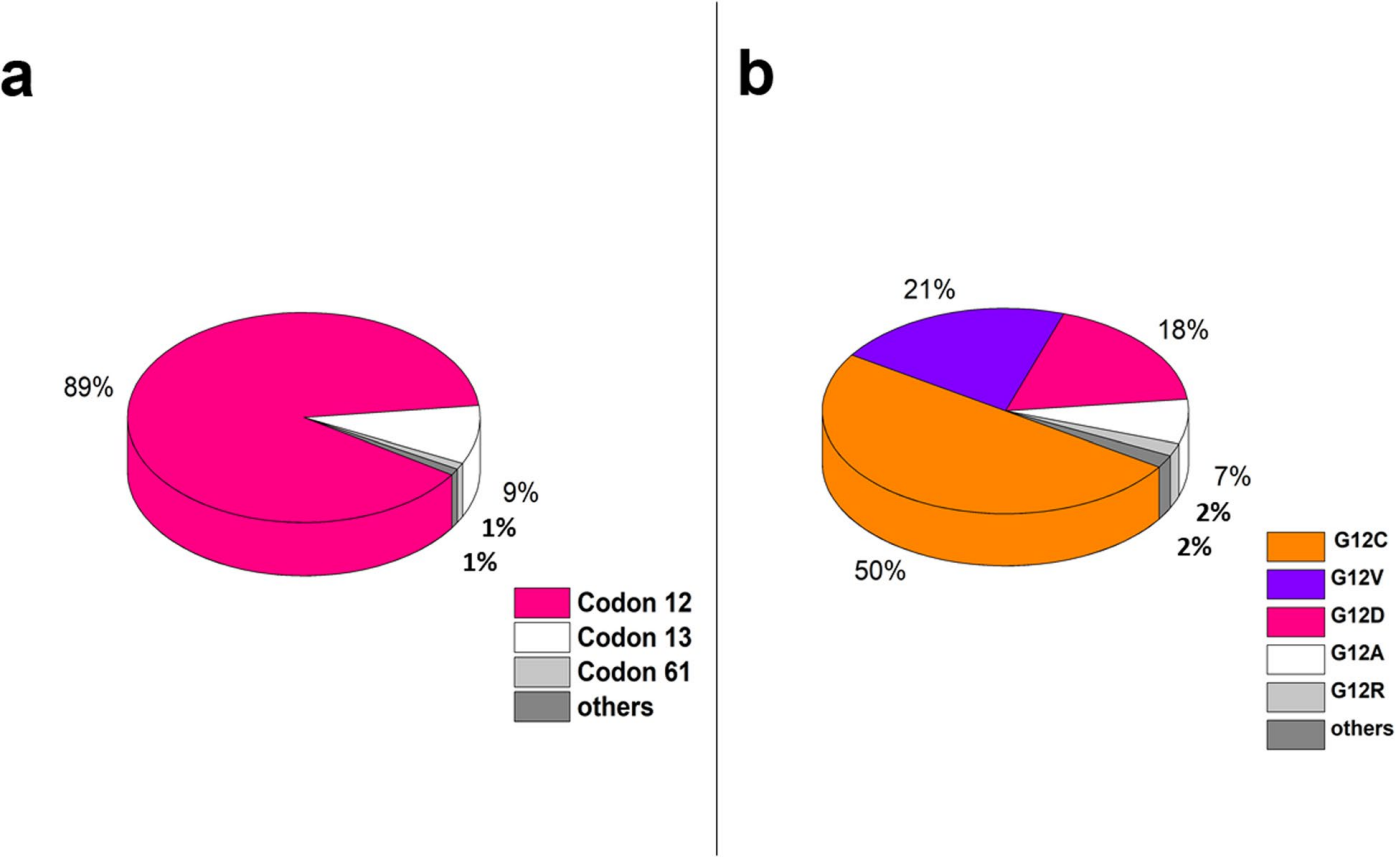
**a** Frequency of occurrence of KRAS mutations in exon 2- codons 12, 13, 61 **b** The spectrum of KRAS mutations in NSCLC, under Codon12, mainly occur as transversions (~ 80%) - G12C, G12V, G12A; G12R. While, transitions occur in ~ 20% cases - G12D, others

KRAS mutations are heterogeneous in their frequency and spectrum in lung cancer and mainly show mutations in codons-12 (89%), 13 (9%), and 61 (1%) [86](Fig. 2).KRAS mutations are categorized into; transitions (a purine-purine, or a pyrimidine-pyrimidine substitution) and transversions (a pyrimidine-purine, or a purine-pyrimidine substitution) [43]. The dominant KRAS mutation patterns are: (i) G → T transversion, in the first base of codon 12(KRAS^G12C^, 40–60%) [87]. In this mutation, glycine is replaced by cysteine and is associated with tobacco smoking [37]. (ii) G → T transversion at the second base of codon 12 replaces glycine by valine (KRAS^G12V^**,** 20–22%) [87, 88]. (iii) G → A transitions at the second base of codons 12 and 13 (KRAS^G12D^ or KRAS^G13D^) are characterized by substitution of glycine with aspartate [89](16–20%) [39]. (iv) G → C transversions at codon 12 with replacement of glycine to alanine, (KRAS^G12A^,7%) or glycine-arginine (KRAS^G12R^,2%) are least frequent.

Regardless of the site of mutation, RAS point mutations lock the mutant RAS into the GTP-bound oncogenic state, encoding oncoproteins, KRAS4A and KRAS4B [90]. Resulting in the accumulation of constitutively GTP-bound RAS proteins inside the cells. KRAS4A expression is specifically expressed in lung epithelial cells while KRAS4B is ubiquitously expressed. Animals lacking KRAS4A have been found to be highly resistant to the development of lung tumor [91]. Thereby, suggesting the essential role of KRAS4A in KRAS-driven lung tumors and its importance in the design and development of KRAS-targeted therapeutics [92]. These mutated RAS proteins bind to RAS effector proteins based on their unique C-terminal hypervariable region and exhibit specific functions [93–95]. The downstream effectors that have been identified include; RAF, MEK, ERK [96], PI3K, AKT, mTOR,Rac1small GTPase and RALGDS/RAL signaling pathways [97, 98](Fig. 1).

Thus RAS oncogenic mutations not only contribute to cancer phenotype, progression and prognosis but are also indicative of their specific downstream signaling pathways (Table 2). For eg. KRAS–G12D preferentially activates AKT signaling whereas KRAS–G12C and G12V preferably activate RAL-A/B signaling [99]. RAF hyperactivation [100] causes MEK1/2 and ERK1/2 phosphorylation and increases their expression in lung cancers [72]. The activated RAS-PI3K-AKT-mTOR pathway promotes cell survival [101] while the activated RAS-RAF-MEK-ERK promotes cell proliferation, survival, and differentiation [102]. These pathways may serve as promising targets to inhibit cancer progression in KRAS mutant lung cancers [96].

## Current molecular methods for KRAS mutation detection

The molecular methods of detecting oncogenic KRAS on clinical samples include: nucleic acid sequencing (Sanger/di-deoxy) [61], pyrosequencing [63], real-time PCR with HRManalysis (high-resolution melting) [103] and allele-specific PCR [104], single nucleotide probe extension assays (SNaPshots) [105], or shifted termination assays (STAs) [106] (Table 2). Useful screening methods include conformation-based separation using single-strand conformation polymorphism (SSCP) and denaturing gradient gel electrophoresis (DGGE). The biggest limiting factors in analysis are; small biopsy size, limited amount of DNA and intrinsic KRAS heterozygous status of tumors, (comprising of mutant and wild-type KRAS).

### Sanger sequencing

Sanger/dideoxy DNA sequencing method is the gold standard to detect KRAS mutations [61] and their potential variations (substitutions, insertions and deletions). It has a high accuracy of ~ 90% but low analytical sensitivity of ~ 85% as compared to higher sensitivity of other methods such as allele-specific PCR, pyrosequencing, and chip array hybridization (90%, 93%, and 92%, respectively) [107]. The Sanger sequencing method requires at least 30%–40% of neoplastic/ non-neoplastic cells to detect mutations [63]. The detection of gain-of-function mutations in KRAS oncogene is a particular challenge, since thetumor cells can carry one copy of wild-type allele and the non-neoplastic cells in tumor tissue can contribute two wild type alleles.

### Whole exome sequencing

Whole exome sequencing (WES) identifies the disease causing variations in protein coding regions of mutated exons in tumor DNA as compared to normal DNA [36, 62, 108]. This method is however limited, if DNA variations are present outside the exon. In this method, whole exome captures and sequencing is performed by using 200 ng of genomic DNA for library preparation. The library is amplified and hybridized to biotinylated oligos specific for exons (baits). The captured libraries are purified using streptavidin magnetic beads and again amplified by PCR. Normalized libraries are pooled and DNA sequenced using paired-end reads and multiplexed. The raw sequence reads are then mapped to the human reference genome. Previous studies have shown NGS sequencing to outperform allele-specific PCR, Sanger sequencing, and pyrosequencing [107]. WES is a cost-effective way of NGS. Using this method, approximately75% of patients received a therapeutic proposal and nearly 23.1% of patients were treated with NGS directed therapy. These included; PI3K/AKT/mTOR inhibitor therapy (27.8%), PARP inhibitors (24.1%), antiangiogenic therapy (21.5%), MEK inhibitor therapy (8.9%) and immunotherapy (6.3%) [108]. However, no differences of progression free survival ratios were observed between patients treated with matched versus standard therapy [108]. Thus, suggesting, the need for multi-omics strategies comprising of circulating cell-free DNA detection, RNA and whole genome sequencing for improving patient outcome.

### Pyrosequencing

This sensitive DNA extension sequencing assay can detect < 5% mutant KRAS alleles among wild-type alleles. It measures the release of pyrophosphate moieties during the incorporation of a specific nucleotide into the synthesized DNA. By using the resulting program, the specific nucleic acid sequence for the target region can be detected [109]. Pyrosequencing provides a sensitive method to detect the mutant KRAS alleles from paraffin-embedded tissue [63]. However this method is not economical owing to expensive equipment.

### PCR and HRM analysis

PCR methods and high resolution melting curve assays provide a cost-effective, sensitive and reliable mutation analysis using low amounts of DNA [110]. They can discriminate between wild-type and mutant gene in DNA isolated from FFPE tissues. Also they can detect mutations in the commonly mutated genes (KRAS, BRAF, PIK3CA, and AKT1) [64]. Therefore, they are highly applicable to large-scale genotyping. HRM utilizes fluorescent probes complementary to the target amplicon. It is faster in contrast to Sanger sequencing [111, 112]. It distinguishes genetic variants by their differences in melting temperature needed to dissociate probe from target leading to the loss of fluorescence [109]. The disadvantages for HRM include: (i) the need for expensive fluorescently labeled probes. (ii) Additional Sanger sequencing to identify the exact mutational status. (iii) Some rare homozygous mutations might not be detected.

### Allele-specific PCR

This common laboratory method characterizes simple genetic variants such as point mutations. It utilizes allele-specific PCR-based K-RAS kits, to detect mutations in KRAS codons 12,13 etc., (G12D, G12V, G12C, G12S, G12A, G12R, G13D). In this method, the targeted alleles are amplified by amplification refractory mutation system (ARMS) and amplification products are detected with Scorpion probes [65]. A fluorescent signal is generated when these probes bind to the PCR amplicon resulting in the separation of the quencher from the fluorophor.

### SNaP shot assay

This multiplexed single nucleotide probe extension assay detects point mutations from very small quantity of DNA [113]. These include; (i) EGFR mutations- c.2573 T > G (L858R), c.2369C > T (T790M); (ii) KRAS mutations- c.34G > T (p.Gly12Cys), c.35G > T (p.Gly12Val); (iii) PIK3CA mutations- c.1624G > A (E542K), c.1633G > A (E545K); (iv) BRAF mutation- c.1799 T > A (V600E). The SNaPshot assay is performed using PCR primers, dNTPs labeled with a differential fluorescence and extension primers and products are resolved on a capillary sequencer. The SNaP Shot assay differs from the shifted termination assays (STA) that are based on primer-extension methods to detect a specific mutation. STA incorporates multiple labeled nucleotides to the detection primer as compared to singly labeled nucleotides incorporation in SNaP Shot assay [114].

### Screening tests

#### Single-strand conformation polymorphism (SSCP)

SSCP is a simple and sensitive assay for detection of SNP, based on the conformation of the single-stranded DNA (ssDNA). Any change in base pairs causes conformational change of the ssDNA and shifts DNA migration under non-denaturing electrophoresis conditions. The separated-out DNA bands are then visualized by incorporating radio-isotopes/ fluorescent dyes/ capillary-based or silver staining [115]. SSCP analysis is used as a screening method to detect point mutations, small deletions and insertions. However, it cannot detect the precise nucleotide change. For this DNA sequencing additionally needs to be performed [116].

#### Denaturing gradient gel electrophoresis (DGGE)

DGGE is another screening method. It separates the PCR products based on sequence differences and the DNA differential denaturing characteristics. In this denaturing gradient gel electrophoresis, PCR products migrate through increasingly higher concentrations of chemical denaturant in the polyacrylamide gel. This results in denaturation of the weaker melting domains of the double-stranded PCR product first and their separation [117].

## KRAS mutation directed lung cancer therapy

The KRAS mutated lung cancers are driven by sustained KRAS expression and signaling. These cancers are commonly associated with resistance to therapy and poor prognosis [118]. They are treated with conventional chemotherapy unlike KRAS-wild type lung cancers, where molecular targeted therapy is available [86]. Presently, there is renewed interest in therapeutic strategies inhibiting the functional output of mutated KRAS [89, 119] (Table 3, Fig. 1). The current approaches include, inhibitors of the: (i) KRAS membrane associations, (ii) KRAS downstream signaling pathways, (iii) KRAS synthetic lethality, (iv) direct targeting of KRAS, (v) immunotherapy and (vi) RAS-regulated metabolic processes [119]. Of these approaches, immunotherapy with immune checkpoint inhibitors in KRAS-mutant NSCLC [83] has been considered as one of the most promising treatment approaches (Table 3).

### KRAS membrane associations

KRAS protein requires membrane localization to become biologically active. Therefore, impairment of KRAS localization can serve as potential target for KRAS-mutant cancers [120]. For membrane localization, the RAS proteins undergo post-translational modificationand lipidation by enzymatic reactions (prenylation by farnesyltransferase [96] or geranylgeranylation via GGTase-I [118]. Initial studies focused on single-stranded DNA (ssDNA); tipifarnib, lonafarnib, salirasib [67, 68, 121] (Table 3). However, alternative lipidation of RAS proteins by GGTases [122] resulted in failure of FTI therapy of KRAS mutated cancers in clinical trials [123]. Recently, modified FTIswhich specifically react with the CAAX motif of KRAS, and block both its farnesylation and geranylgeranylation are being studied [124]. Another approach to prevent the membrane localization of KRAS protein is by inhibiting the phosphodiesterase 6 delta subunit (PDEδ). Since, PDEδ acts by binding to the farnesylated tail of KRAS and chaperoning its membrane localization [125, 126]. The PDEδ inhibitors can be used to disrupt KRAS:PDEδ binding and disrupting the localization of KRAS in cancer cells [69–71].

### KRAS downstream signaling pathways

RAF, MEK, PI3K, mTOR are some of the downstream effectors of KRAS signaling. Their inhibitors are used as single agents or as combination therapy for treatment of lung cancer. The single agent therapies available for KRAS mutated LADCs are; BRAF inhibitor (Sorafenib) [73], MEK inhibitors (Selumetinib) [74], mTOR inhibitor (ridafarolimus) [75], focal adhesion kinase inhibitor (defactinib) [76]. However, these have shown disappointing clinical efficacy, so far. Recently, a RAF/MEK inhibitor (RO5126766) has shown to effectively reduce tumours in 60% of patients with a low frequency of higher grade adverse events [127]. The combination therapies inhibit two or more downstream effectors in the RAS pathway. They have been observed to fully block KRAS signaling in several phase I trials of lung cancer [11, 12, 128, 129]. However, their phase III validation studies are awaited or have shown failure. For eg. the Selumetinib and docetaxel combination therapy for KRAS-G12C and KRAS-G12V tumours [98, 130] have failed validation in phase III (SELECT-1) [131]. Similarly phase II trials using combination of a PI3K inhibitor (BKM120) with MEK1/2 inhibitor (MEK162) in patients with NSCLC, has shown little success [11, 72].

### KRAS synthetic lethality

The KRAS synthetic lethality approach involves the selective killing of KRAS mutated cancer cells by inhibition of a second protein. In every case, the interactions between mutated KRAS and other proteins on which KRAS mutated cancer cells have become dependent need to be identified first [132]. Then these second hits can be therapeutically targeted resulting in selective death of KRAS-mutant, but not KRAS-wild-type, cells [84]. These include; (i) GATA2 transcription factor (proteasome upregulator) and its inhibitor, bortezomib, which has shown response in KRAS-G12D lung cancers [77]. (ii) Cyclin-dependent kinase 4 (Cdk4) (G1 transition/cell cycle progressor) and its inhibitor proteins, p16^INK4A^, p15^INK4B^, p18^INK4C^ and p19^INK4D^, cause KRAS mutated lung cells to undergo senescence and prevent tumor growth^128^ (iii) TANK-binding kinase 1 (TBK1), serine-threonine kinase STK33 and polo-like kinase 1(PLK1) are other potential synthetic lethal therapeutic targets that have been identified in cell lines [79–81]. Some of these encode protein kinases and may be inhibited by selective TKIs.

### KRAS direct targeting

RAS has been perceived to be “undruggable” due to its lack of deep pockets for binding of small molecule inhibitors. However, recent studies have shown some success in the direct inhibition of RAS [120, 133]. This strategy targets the RAS proteins activation and prevents the conversion of inactive KRAS to active KRAS. These include molecules that can, (i) allosterically bind to the Switch-II pocket in GDP-RAS, adjacent to the cyteine residue of KRAS-G12C [134, 135]. (ii) directly bind and impair wild-type KRAS activation by the SOS1-GEF [136]. (ii) selectively recognize and inactivate specific KRAS G12C amino acid substitution [137]. Their results are a majorstep forward in the development of direct KRAS^G12C^ inhibitor therapy for lung cancer [133].

The Ras GTPase family inhibitor, sotorasib has recently been approved by US-FDA for treatment of KRAS mutated locally advanced or metastatic solid tumours (NSCLC and colorectal cancer) [138]. Simultaneous approval has been given to the QIAGEN *therascreen®* KRAS RGQ PCR kit (tissue) and the Guardant360® CDx (plasma) as companion diagnostics. They have recommended that the tumor tissue should be tested if no mutation is detected in plasma specimen. Previously in the clinical trial of sotorasib conducted by Hong et al., 2020 a confirmed objective response in 32.2% patients and disease control (stable disease) in 88.1% NSCLC patients [139] was observed. Adagrasib (MRTX849) is another novel small molecule targeting the KRAS^G12C^ mutation that has shown promising activity [140].

However, the mechanisms of acquired resistance to these therapies are currently unknown. Using in vitro deep mutational screening methods diverse genomic and histologic mechanisms imparting resistance to KRAS^G12C^ inhibitors have been identified [140]. These acquired KRAS alterations include G12D/R/V/W, G13D, Q61H, R68S, H95D/Q/R, Y96C, and high-level amplification of the KRASG12C allele. Additionally, there exist acquired bypass mechanisms of resistance; (i) MET amplification, (ii) activating mutations in NRAS, BRAF, MAP 2 K1, and RET; (ii) oncogenic fusions involving ALK, RET, BRAF, RAF1, and FGFR3; (iii) and loss-of-function mutations in NF1 and PTEN. These secondary KRAS mutations can cause resistance to sotorasib, adagrasib, or both, in vitro and are suggestive of their sequential use [141]

### Immunotherapy

KRAS mutant lung cancers have an immune resistant microenvironment. The immune resistance is caused by smoking that induces T-cell influx and PD-L1 expression by cancer cells and tumor infiltrating lymphocytes (TILs) [119]. PD-L1 positivity is seen in approximately 60–70%TILsand in 20–55%KRAS-mutant tumor cells [142–144]. Therefore immune checkpoint inhibitor therapy is being investigated to improve the patient outcome in KRAS mutant cancers [83]. Previously, Falk et al., 2016, have observed hypoxia to significantly increase the PD-L1 expression in KRAS^G12C^ and KRAS^G12D^ codon subtypes [145]. Identification of the coexisting KRAS mutations subtype may serve as biomarkers of resistance and need to be performed prior to initiation of PD-1/PD-L1 inhibitor therapy [83].

### Inhibition of RAS-regulated metabolic processes

Cancer cells harboring KRAS mutation show up-regulation of rate-limiting enzymes, shift of cancer cell metabolism toward anabolic pathways resulting in increased cancer cell growth. Therefore, recent studies are focusing on mutant KRAS-driven metabolic rewiring, including; (i) upregulation of enzymes involved in amino acid, fatty acid or nucleotide biosynthesis. Glucose and glutamine metabolism,(ii) deregulation of scavenging cellular pathways (e.g., autophagy) [146], (iii) PPARγ and WNT/β-catenin, pathways involved in metabolic enzymes changes in cancers [147, 148]. Currently, the metabolic dependencies of oncogenic KRAS driven lung and pancreatic cancers are still in their infancy and hold promise as therapeutic targets [149].

## Conclusion

The KRAS driven lung cancers can be categorized into different subsets (such as KL, KP, KC, etc). This categorization is based on tumour histology, type of KRAS mutation (transversions/transitions) and presence of co-existing significant mutations in nearly eighteen genes identified so far. KRAS mutations have heterogeneous spectrum in lung cancer- transversions (80%)/ transitions (20%). KRAS mutations correlate with histology: transversions, more frequently develop adenocarcinoma while transitions more frequently have squamous cell carcinoma. These biologically distinct subsets have unique intracellular signaling patterns and are susceptible to different therapeutic strategies. Thus these subsets can be used to predict new targeted therapeutic strategies and improve the prognosis of lung cancer patients.

A variety of molecular methods are now available for detecting oncogenic KRAS in clinical samples. These entail complete molecular profiling of each patient and identification of KRAS mutations and coexisting mutations. The biggest limiting factors in analysis are; small biopsy size, limited amount of DNA and intrinsic KRAS heterozygous status of tumors.

The G → T transversion, in the first base of codon 12 (KRAS^G12C^) mutation is the commonest in lung adenocarcinomas. Presently, there is renewed interest in therapeutic strategies inhibiting the functional output of mutated KRAS. Direct KRAS^G12C^ inhibitor therapy and immunotherapy with immune checkpoint inhibitors are being considered as one of the most promising treatment approaches. These may prove to be a step forward in personalized therapy and in improving prognosis of lung cancer patients.

## Data Availability

Not applicable.

## Abbreviations

ARID1A: AT-Rich Interaction Domain 1A
BRAF: B-Raf Proto-Oncogene
CCL-9: Chemokine (C-C motif) ligand 9
CD74: ROS1-Cluster of Differentiation 74-ROS proto-Oncogene 1
CDKN2A: Cyclin-dependent kinase inhibitor 2A
DGGE: Denaturing gradient gel electrophoresis
dNTPs: dideoxy nucleotides
EGFR: Epidermal growth factor receptor
EML4: ALK-Echinoderm microtubule-associated protein-like 4-anaplastic lymphoma kinase
ERBB2: v-erb-b2 avian erythroblastic leukemia viral oncogene homology 2
ERK: Extracellular signal-regulated kinase
GEF: Guanine Exchange Factors
GTP: Guanosine triphosphate
HRAS: Harvey rat sarcoma viral oncogene
HRM: High-resolution melting analysis
HSP-90: Heat Shock Protein-90
IFNγ: Interferon gamma
IL-23: Interleukin-23
KEAP1: Kelch-like ECH-associated protein 1
KIF5B-RET: Kinesin family member 5B-Ret proto Oncogene
KRAS: Kirsten rat sarcoma virus transforming protein
LADC: Lung adenocarcinoma
MEK: Mitogen extracellular signal-related kinase
MET: Mesenchymal epithelial transition factor
mTOR: Mammalian Target of Rapamycin
MYC: MYC proto Oncogene
NF1: Neurofibromin 1
NF-κB: Nuclear Factor kappa-light-chain-enhancer of activated B cells
NRAS: Neuroblastoma Ras virus oncogene
NRB1: Neurabin1
NSCLC: Non-small cell lung cancer
NTRK: Neurotrophilic tyrosine receptor kinase
PI3K: Phosphatidylinositol-4,5-bisphosphate 3-kinase
PPARγ: Peroxisome Proliferator Activated Receptor Gamma
Rac1small GTPase: Ras-related C3 botulinum toxin substrate 1
RAF: Rapidly accelerated fibrosarcoma
RALGDS/RA: Ral guanine nucleotide dissociation stimulator
RB1: Retinoblastoma 1
RIT1: Ras-like protein in tissues 1
SETD2: SET Domain Containing 2
SSCP: Single-strand conformation polymorphism
ssDNA: Single-stranded DNA
STK11: Serine/threonine kinase 11
TP53: Tumour protein 53
TTF1: Transcription Termination Factor 1
WNT/β-catenin: Wingless and Int-1/ beta-catenin
